# Determinants of health expenditure in OECD countries: A decision tree model

**DOI:** 10.12669/pjms.336.13300

**Published:** 2017

**Authors:** Nesrin Akca, Seda Sonmez, Ali Yilmaz

**Affiliations:** 1 Nesrin Akca, PhD.Department of Health Management, Faculty of Health Sciences, Kirikkale University, Kirikkale, Turkey; 2 Seda Sonmez, MSc.Department of Health Management, Faculty of Health Sciences, Kirikkale University, Kirikkale, Turkey; 3 Ali Yilmaz, PhD.Department of Health Management, Faculty of Health Sciences, Kirikkale University, Kirikkale, Turkey

**Keywords:** Classification, Data mining, Decision tree method, Health expenditure, OECD

## Abstract

**Objective:**

This study aimed to identify the major variables in the estimation of health expenditure in OECD member countries with the decision tree method and to categorize the member countries by health expenditure.

**Methods:**

The study population comprised the 2014 data of the 35 OECD countries. In the study, health expenditure as a share of gross domestic product was the dependent variable while gross domestic product per capita, percentage of total population covered by public and private insurance, out-of-pocket health expenditure as percentage of total expenditure on health, age dependency ratio, life expectancy at birth, number of hospitals per million population, number of physicians per 1000 population/head counts, pharmaceutical sales and perceived health status were designated as independent variables. The decision tree model was constructed with the CART algorithm using the Orange data mining software package.

**Results:**

In the study, GDP per capita, life expectancy at birth, age dependency ratio, number of hospitals and percentage of the population with a bad perceived health status were identified as the major variables in the estimation of health expenditure. OECD countries were categorized in 6 groups according to the decision tree model. According to the CART algorithm used in the model, the classification accuracy rate and the precision of estimation were computed as 80.56% and 81.25%, respectively.

**Conclusion:**

The study results revealed that the most important determinant for estimating the share of GDP allocated to health expenditure was GDP per capita. Future studies should be conducted with the inclusion of different variables in the model.

## INTRODUCTION

Open systems such as healthcare organizations are significantly affected by changes in their internal and external environment.^1^ Population aging and the resulting rise in chronic diseases, medicinal and technological developments, changing competition trends in the healthcare market, healthcare legislation and changing healthcare workforce structure have led to increased health expenditures.^2^ As countries combat increasing health costs, they also try to ensure access to comprehensive and non-discriminatory healthcare services and to protect their citizens from intolerably high healthcare costs.^3^ In OECD (Organization for Economic Co-operation Development) countries, health spending as a share of GDP rises yearly.^4^ Projections indicate that this increase will endure in the next 50 years (in the study, 2010 data were used in the estimation) and that health expenditure for OECD countries will reach approximately 14% in 2060 but that it could be reduced down to 9.5% with regulations in national healthcare policies.^5^ The level of national health expenditure is a major issue for developed, developing and undeveloped countries. The correct question needs to be formulated in order to determine what a country’s health expenditure should be: “How much should my country spend on health, given our current epidemiological profile relative to our desired level of health status, considering the effectiveness of health inputs that would be purchased at existing prices, and taking account of the relative value and cost of other demands on social resources?”.^6^ Therefore, determination of a country’s health expenditure is a process that requires consideration of several factors.

Due to its constant increase, investigation of the determinants of health expenditure has become one of the major issues for health policy makers and planners.^7^ Numerous studies aimed at identifying these determinants have been carried out. Past studies have identified revenue (GDP) as indubitably the most important determinant of health expenditure.^8^ Other variables considered to be determinants of health expenditure are age dependency ratio and epidemiological needs, advancements in medical technology, health system characteristics, out-of-pocket health expenditures, population disease pattern, health insurance system, number of physicians, number of prescribed drugs per person, number of hospitals, crude birth rate, literacy rate and life expectancy at birth.^9^-^14^

This study aimed to identify the major variables in the estimation of health expenditure in OECD member countries and to categorize the member countries by these variables. The facts that the study introduces a different perspective and the results support similar studies in the literature highlight its significance. In order to estimate the determinants of health expenditure in OECD countries, Phi (2017)^15^ used panel data analysis to investigate the 2000-2013 data and found similar results.

## METHODS

This study aimed to identify the major variables in the estimation of the share of gross domestic product allocated to health expenditure in OECD member countries and to categorize the member countries by these variables with the decision tree method.

The study population comprised data from the 35 OECD member countries. In the study, the distribution of the share of GDP allocated to health expenditure was examined. The member countries were classified in two categories as those with GDP shares below or above the mean value of 9% (8.97%)^4^ and this was used as a dependent variable in the analysis. With a review of literature, the variables considered to act on health expenditure were identified and employed as independent study variables. Gross domestic product per capita, percentage of total population covered by public and private insurance, out-of-pocket health expenditure as percentage of total expenditure on health, age dependency ratio, life expectancy at birth, number of hospitals per million population, number of physicians per 1000 population/head counts, perceived health status and pharmaceutical sales were designated as independent variables (Table-I). Only 2014 data were included in the study as the unavailability of a complete set of data for all study variables could impact the model. The variables were selected after a review of literature and peer debriefing.

**Table-I:** Decision Tree Model Variables.

*Dependent Variable*	*Data Source*
Health expenditures as a share of gross domestic product (%)	OECD
Independent Variables	
Gross domestic product per capita (USD, current PPPs)	OECD
Out-of-pocket health expenditure (% of total expenditure on health)	World Bank
Age dependency ratio (%of working age population)	World Bank
% of total population covered (public and private)	OECD
Life expectancy at birth (years)	OECD
Number of hospitals (per million population)	OECD
Number of physicians (density per 1000 population/head counts)	OECD
Pharmaceutical sales (millions of national currency units)	OECD
Perceived health status (good, fair and bad-% of population)	OECD

Orange data mining software package Version 2.7 was used in data analysis. In order to determine the algorithms to be used in the decision tree model, the performance criteria for ID3, C4.5 and CART algorithms – the most prevalent decision tree algorithms – were evaluated with 10-fold cross validation. In addition, the target class was designated as “equal to or below 9%” (coded as 0 in the model) in all decision tree algorithms. The criteria used for analyzing algorithm performance were classification accuracy rate, sensitivity, specificity, the area under the ROC curve, information score, F1 score, precision, recall, Brier score and Matthews Correlation Coefficient. According to these criteria, the algorithm with the best performance was identified as the CART algorithm (Table-II) and the decision tree model was constructed with this algorithm by the criteria of a minimum of 5 elements in a node, the tests conducted are illustrated in Table-II.

**Table-II:** Performance Criteria for Decision Tree Algorithms (10-fold cross validation).

*Algorithms / Performance Criteria*	*CA*	*Sens*	*Spec*	*AUC*	*IS*	*F1*	*Prec*	*Recall*	*Brier*	*MCC*
ID3	0.7444	0.7647	0.7222	0.7500	0.4764	0.7429	0.7222	0.7647	0.4550	0.4869
C4,5	0.8056	0.7647	0.8333	0.7407	0.5245	0.7879	0.8125	0.7647	0.3993	0.6000
CART	0.8056	0.7647	0.8333	0.7870	0.5595	0.7879	0.8125	0.7647	0.3704	0.6000

CA: Classification Accuracy Sens: Sensitivity Spec: Specificity AUC: Area Under the ROC Curve IS: Information Score F1: F1 Score (harmonic mean of precision and recall) Prec: Precision Recall: Recall Score Brier: Brier Score MCC: Matthew Correlation Coefficient

## RESULTS

Descriptive statistics for the dependent and the independent variables (Table-I) of the model are presented in Table-III. The table shows a 9% mean share of GDP allocated to health expenditure in OECD countries in 2014. The means for GDP per capita, out-of-pocket health expenditure as a share of overall health spending, age dependency ratio, percentage of population with health insurance, life expectancy at birth, number of hospitals per million population, number of physicians per 1000 population/head counts and pharmaceutical sales were 40081.29 USD, 19.62%, 51.17%, 98.15%, 80.61 years, 29.71 hospitals, 4.65 physicians and 1126244.355 units; respectively. The mean percentages of the population with good, fair and bad perceived health status^4^ (used as characterized in the OECD database) were 69.91%, 21.39% and 9.35%; respectively.

**Table-III:** Descriptive Statistics for Model Variables.

*Variables*	*Short Name*	*Min*	*Max*	¯X	*SD*
Health expenditures as a share of gross domestic product	HE	5.09	16.59	8.97	2.26
Gross domestic product per capita	GDP	17973.26	100052.81	40081.29	15403.68
Out-of-pocket health expenditure	OOP	5.22	44.00	19.62	9.03
Age dependency ratio	AGE	36.97	63.41	51.17	5.62
% of total population covered	PPC	88.50	100.00	98.15	3.17
Life expectancy at birth	LEB	74.30	83.70	80.61	2.52
Number of hospitals	NHC	10	73	29.71	15.26
Number of physicians	NPC	2.02	7.32	4.65	1.32
Pharmaceutical sales	PS	191.30	23665459.10	1126244.355	4507036.39
Perceived health status (good)	PHSG	32.50	91.40	69.91	13.31
Perceived health status (fair)	PHSF	6.80	51.60	21.39	9.22
Perceived health status (bad)	PHSB	1.70	18.30	9.35	4.65

**Fig.1:**
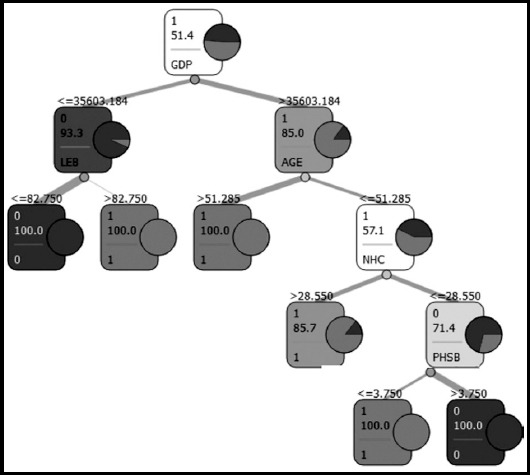
Decision Tree Graph

The decision tree model constructed with the CART algorithm revealed GDP per capita as the most important determinant for estimating the share of GDP allocated to health expenditure. The other important variables were life expectancy at birth, age dependency ratio, number of hospitals and percentage of the population with a bad perceived health status (Fig.1).

According to the result of the analysis, the 35 OECD member countries were categorized in 6 groups: (Fig.1)

1. Countries with a GDP per capita under 35603.184 USD and a life expectancy at birth equal to or less than 82.75 years
2. Countries with a GDP per capita under 35603.184 USD and a life expectancy at birth more than 82.75 years
3. Countries with a GDP per capita over 35603.184 USD and an age dependency ratio greater than 51.285%
4. Countries with a GDP per capita over 35603.184 USD, an age dependency ratio equal to or less than 51.285% and the number of hospitals per million population more than 28.550
5. Countries with a GDP per capita over 35603.184 USD, an age dependency ratio equal to or less than 51.285%, the number of hospitals per million population equal to or less than 28.550 and population percentage with a bad perceived health status equal to or less than 3.750%,
6. Countries with a GDP per capita over 35603.184 USD, an age dependency ratio equal to or less than 51.285%, the number of hospitals per million population equal to or less than 28.550 and population percentage with a bad perceived health greater than 3.750%.

## DISCUSSION

Health policy makers and planners strive to develop better solutions for the management of health expenditure that continues to increase throughout the globe. Fair and equal allocation of limited resources to the society is an endeavor that requires planned and organized execution. Therefore, policy makers need evidence-based data to build their decisions upon. In OECD member countries, health expenditure rises every year. In the study, the major variables in the estimation of the share of gross domestic product allocated to health expenditure in OECD member countries were identified and the member countries were categorized by these variables with the decision tree method.

In the study, the most significant determinant in the estimation of the share of gross domestic product allocated to health expenditure was determined as GDP per capita. The other important variables were found to be life expectancy at birth, age dependency ratio, number of hospitals and percentage of the population with a bad perceived health status, and OECD member countries were categorized in 6 groups. The study by Okunade (2005) conducted to identify the determinants of health expenditure in African countries reported GDP (elasticity estimate of about 0,65 in relation to health expenditure) as the most important determinant.^16^ The study carried out by Di Matteo (2005) to investigate the macro determinants of health expenditure in the United States and Canada with respect to income, age distribution and time revealed a significant relationship of health expenditure with aging population, income and time. In the models constructed for USA and Canada, total impact and contribution of age and time on health expenditure were found to be similar, with time and 65 years-old and older age group variables explaining 71% and 75% of the increase in personal health expenditure in USA and Canada, respectively.^17^ Toor and Butt (2005) reported per capita GDP, urbanization, literacy rate, crude birth rate and foreign aid as important determinants of health expenditure in Pakistan. According to the regression model, these variables explained 99% of the variation in health expenditure.^13^ The study by Hosoya (2014) investigating the determinants of health expenditure in OECD countries identified age, GDP, labor force participation rate of women, population, unemployment rate and time as the major determinants of health expenditure. The study also found an income elasticity of smaller than 1, indicating that healthcare service is not a luxury but an imperative necessity.^18^ Bradley et al. (2011) aimed to examine the association of health and social services expenditures in OECD countries with population-level health outcomes, i.e. life expectancy at birth, infant mortality, low birth weight, maternal mortality and potential years of life lost. They found that expenditure for health services was associated with better health outcomes (only life expectancy at birth (p<0,001) and potential years of life lost (p=0,02)).^14^ Furuoka et al. (2011) reported GDP and aging population as the two major variables of health expenditure in Asian countries. They reported that the two-way fixed-effect model was the most appropriate model among the ones constructed in the study and that this model explained 99% of the variation in health expenditure.^19^ In order to estimate the determinants of health expenditure in OECD countries, Phi (2017) used panel data analysis to investigate the 2000-2013 data. The study adopted health expenditure per capita as the dependent variable and health expenditure per capita as a share of GDP per capita, GDP per capita, public financing, number of physicians per 1000 population, number of hospital beds per 1000 population, alcohol consumption, tobacco consumption, life expectancy and population > 65 years old as the independent variables. All the models constructed in the study yielded similar results where GDP elasticity was smaller than 1, emphasizing that healthcare service is not a luxury. The study results showed GDP, life expectancy and population aged 65 years and older as the most important determinants of health expenditure in OECD countries.^15^

In conclusion, the results of the present study are congruent with past studies. Furthermore, the present study identified health outcomes (i.e. life expectancy at birth and percentage of population with bad health perception) as determinants of health expenditure alongside GDP and age. We believe our findings will contribute to the literature and provide important evidence-based information to health policy makers and planners. However, as the factors that act on health expenditure are not limited to the variables of the present study, investigating other factors in future studies would provide more comprehensive information.

### Authors’ Contribution

**Nesrin Akca:** Preparation of final manuscript.

**Seda Sonmez:** The data was analyzed.

**Nesrin Akca, Seda Sonmez and Ali Yilmaz:** The literature research was done by all authors.
